# Pharmacokinetic Analysis of Diosgenin in Rat Plasma by a UPLC-MS/MS Approach

**DOI:** 10.1155/2022/5607347

**Published:** 2022-09-30

**Authors:** Pei Liu, Lin Xu, Jing-han Guo, Jin-hua Chang, Xi-gang Liu, He-fei Xue, Ru-xing Wang, Zhong-si Li, Guang-xin Miao, Cui-zhe Liu, Jian-yu Zhou

**Affiliations:** ^1^Hebei Province Key Laboratory of Nerve Injury and Repair, Hebei Province Key Laboratory of Research and Development for Chinese Medicine, Chengde Medical University, Chengde, Hebei 067000, China; ^2^Beijing North Institute of Biotechnology, No. 20 Panjiamiao Rd, Fengtai District, Beijing 100071, China

## Abstract

Diosgenin, a steroidal sapogenin, has attracted attention worldwide owing to its pharmacological properties, including antitumor, cardiovascular protective, hypolipidemic, and anti-inflammatory effects. The current diosgenin analysis methods have the disadvantages of long analysis time and low sensitivity. The aim of the present study was to establish an efficient, sensitive ultrahigh-performance liquid chromatography-tandem mass spectrometry (UPLC-MS/MS) approach for pharmacokinetic analysis of diosgenin amorphous solid dispersion (ASD) using tanshinone IIA as an internal standard (IS). Male Sprague-Dawley rats were orally administered diosgenin ASD, and orbital blood samples were collected for analysis. Protein precipitation was performed with methanol-acetonitrile (50 : 50, v/v), and the analytes were separated under isocratic elution by applying acetonitrile and 0.03% formic acid aqueous solution at a ratio of 80 : 20 as the mobile phase. MS with positive electron spray ionization in multiple reaction monitoring modes was applied to determine diosgenin and IS with *m/z* 415.2⟶271.2 and *m/z* 295.2⟶277.1, respectively. This approach showed a low limit of quantification of 0.5 ng/ml for diosgenin and could detect this molecule at a concentration range of 0.5 to 1,500 ng/ml (*r* = 0.99725). The approach was found to have intra- and inter-day precision values ranging from 1.42% to 6.91% and from 1.25% to 3.68%, respectively. Additionally, the method showed an accuracy of -6.54 to 4.71%. The recoveries of diosgenin and tanshinone IIA were 85.81–100.27% and 98.29%, respectively, with negligible matrix effects. Diosgenin and IS were stable under multiple storage conditions. Pharmacokinetic analysis showed that the *C*_max_ and AUC_0⟶t_ of diosgenin ASD were significantly higher than those of the bulk drug. A sensitive, simple, UPLC-MS/MS analysis approach was established and used for the pharmacokinetic analysis of diosgenin ASD in rats after oral administration.

## 1. Introduction

**Figure 1:**
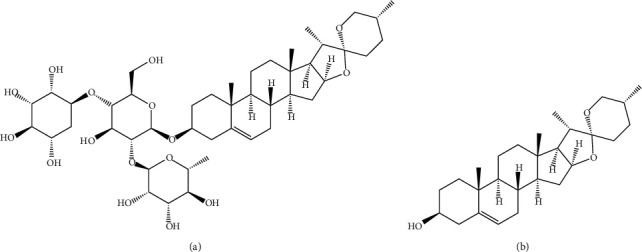
The structural formulas of (a) dioscin and (b) diosgenin.

Dioscin is a steroid saponin that is mainly present in the families *Dioscoreaceae*, *Liliaceae*, and *Caryophyllaceae* and is the most abundant in Dioscorea zingiberensis C. H. Wright, Dioscorea nipponica Makino, and other *Dioscoreaceae* [1]. Moreover, this chemical is the major active constituent of several Chinese patent medicines, including Liuwei Dihuang pills, Wei Aoxin tablets, and Di'ao Xin Xue Kang capsules [2–4]. Research has shown that dioscin is mainly metabolized to diosgenin (Figure 1) through the gut microbiota *in vivo* [5, 6]. Diosgenin has attracted attention worldwide owing to its pharmacological effects, including antitumor [7], cardiovascular protective [8, 9], hypolipidemic, and anti-inflammatory effects [10]. Currently, the antitumor efficacy of diosgenin has attracted the most attention. Indeed, diosgenin exhibits antitumor effects in a variety of cancer cells, particularly liver cancer cells [11, 12], through immunomodulatory effects and enhancement of the gut microbiota [13]. Diosgenin also prevents atherosclerosis through its anti-inflammatory actions [14], thereby preventing hypercholesterolemia [15] and blocking the expression of the Notch signaling pathway. In addition, diosgenin improves testicular injuries in streptozotocin-diabetic rats by attenuating apoptosis, oxidative stress, and inflammation [16].

Diosgenin may have potential clinical applications. Nevertheless, like many new compounds with therapeutic attributes, diosgenin has some disadvantageous characteristics such as poor aqueous solubility [17, 18]. The strong hydrophobicity of diosgenin results in low bioavailability [19]. According to pharmacokinetic analyses, the absolute bioavailability of diosgenin in rats is only approximately 7% [20]. Hence, a simple and effective approach should be developed to enhance the bioavailability of diosgenin. At present, research focuses on amorphous solid dispersions (ASDs) for improvement of the dissolution and solubility of poorly water-soluble compounds [21–23]. In our previous study, ASD technology was successfully used to improve the solubility and bioavailability of diosgenin using a coprecipitation method.

Our knowledge of the important role of diosgenin is based on the pharmacokinetic study of this compound in animals. Gas chromatography-mass spectrometry (GC-MS), and high-performance liquid chromatography tandem mass spectrometry (HPLC-MS/MS) have been used to detect and analyze diosgenin. However, these reported approaches have some disadvantages, including long analysis time, weak sensitivity, and high cost [24–29]. From this perspective, a convenient, sensitive, simple, and low-cost analytical approach is needed to assess diosgenin quickly. With advancements in technology, ultrahigh-performance liquid chromatography tandem mass spectrometry (UPLC-MS/MS) has been used to analyze many chemicals. This method shows faster analysis, better sensitivity, and more precise resolution than those of HPLC–MS/MS; hence, UPLC-MS/MS has become the preferred method for analysis and detection, enabling substantial progress in environmental and biological analyses and pharmaceutical research [30–32].

Accordingly, in this study, we developed a new UPLC-MS/MS approach to quantify diosgenin in rat plasma. This approach presents several advantages, including short analysis time, high sensitivity, excellent reproducibility, and easy sample processing, and could be used for pharmacokinetic research on diosgenin ASD to provide insights into rational clinical use.

## 2. Materials and Methods

### 2.1. Chemicals and Reagents

Diosgenin was purchased from Nanjing Spring & Autumn Biological Engineering Co., Ltd. (Nanjing, China) and had a purity of >98% (lot number: 20180731). The National Institute for Food and Drug Control (Beijing, China) provided the chemical criteria for diosgenin (lot number: 111539–200001) and tanshinone IIA (used as an internal standard (IS), purity >98%, lot number: 110766–200518). BASF Co., Ltd. (Shanghai, China) provided Soluplus (lot number: 20747936W0). MS-grade acetonitrile, methanol, and formic acid were purchased from Thermo Fisher Scientific (Tustin, CA, USA, lot number: 211511, 182084, 179246). Ultrapure water was obtained from A.S. Watson Group (Hong Kong) Ltd (lot number: 32834144858).

### 2.2. Preparation of Diosgenin ASD

Diosgenin and Soluplus were mixed and dissolved in anhydrous ethanol at a w/w ratio of 1 : 10. The organic solvent was dried using a rotary evaporator. The product was frozen for 30 min and then vacuum-dried (DZ-2BCIV, Tianjin Tester Instrument Co., Ltd., Tianjin, China). The experiment was repeated three times to obtain diosgenin ASD.

### 2.3. UPLC-MS/MS Conditions for Analysis of Diosgenin

Liquid chromatography was performed using a UPLC system (Waters Corp., Milford, MA, USA) interfaced to a Q-Trap 5500 triple-quadruple mass spectrometer (AB SCIEX, Beijing, China) equipped with an electrospray ionization (ESI) source, as previously described [33]. The isolation was performed on a Waters ACQUITY UPLC BEH C_18_ rapid resolution high definition column (2.1 × 50 mm, 1.7 *μ*m) at 35°C. The temperature of the UPLC autosampler was 4°C. The mobile phase was mixed with acetonitrile and water (80 : 20, v/v, containing 0.03% formic acid in water). The flow rate was run at 0.3 ml/min. The injection volume was 2 *μ*l, with a total running time of 5 min.

**Figure 2:**
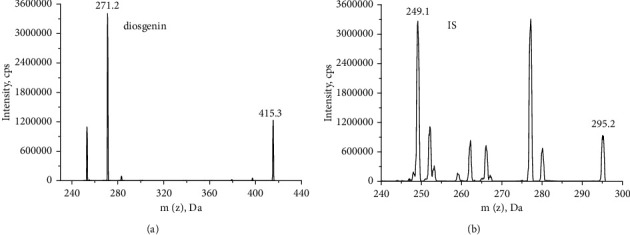
The mass spectra of (a) diosgenin and (b) IS.

The positive ion multiple reaction monitoring (MRM) mode was used for quantification analysis in the mass analyzers, with the transitions of *m/z* 415.2⟶271.2 for diosgenin with fragment or voltage of 71.96 V and 295.2⟶277.1 for the IS with fragment or voltage of 120 V. The collision voltages for diosgenin and IS were 23.03 and 25.92 eV, respectively. The other settings were as follows: capillary voltage, 5.5 kV; source temperature, 500°C; curtain gas, 35 Psi; GS_1_, 50 Psi; GS_2_, 50 Psi. The mass spectra of diosgenin and IS are shown in Figure 2.

### 2.4. Preparation of diosgenin standard solution and quality control (QC) samples

Standard working solutions of diosgenin and IS were prepared by diluting stock solutions (100 *μ*g/ml) with methanol. A series of calibration standards and QC working solutions were serially diluted with methanol from the standard solution. The working solutions were diluted with drug-free rat plasma at concentrations of 0.5, 1, 5, 50, 200, 1000, and 1,500 ng/ml. Similarly, the QC samples were made up at 5, 50, and 1,000 ng/ml, and IS was 42 ng/ml. All samples were immediately stored at −20°C.

### 2.5. Plasma Sample Preparation

Blank plasma samples were obtained from control Sprague-Dawley rats (*n* = 6). Briefly, 50 *μ*l of the IS solution and 50 *μ*l of plasma were added to a 0.5-ml Eppendorf tube and vortex mixed for 30 s. Methanol-acetonitrile (250 *μ*l; 50 : 50, v/v) was added to the Eppendorf tube, and then, the sample was vortex mixed for 2 min. After centrifuging at 15,000 rpm for 15 min, the supernatants were injected into the UPLC-MS/MS system.

### 2.6. Method Validation

A series of confirmatory experiments following the guidelines of the ARRIVE, the Food and Drug Administration (FDA), and the Pharmacopeia of the People's Republic of China based on the validation of bioanalytical assays [34–36] was conducted, characterizing the specificity, linearity, LLOQ, accuracy, precision, matrix effects, recovery, and stability of the method.

### 2.7. Specificity

The specificity was evaluated by comparing the chromatograms of blank plasma and related spiked plasma. This was performed to ensure that endogenous and other compounds in the sample could not interfere with diosgenin and IS analysis.

### 2.8. Linearity and LLOQ

An aliquot of the working solution (50 *μ*l, at various concentrations), IS solutions (50 *μ*l, 42 ng/ml), and 200 ml methanol-acetonitrile (50 : 50, v/v) were mixed to blank rat plasma (50 *μ*l) independently to obtain calibration samples. Afterward, the final concentration range of diosgenin was 0.5–1,500 ng/ml. The calibration curves for diosgenin were established with the concentrations (0.5, 1, 5, 50, 200, 1000, and 1,500 ng/ml) and using 1/*X*^2^ to plot the peak area ratio (diosgenin/IS, *Y*) versus the concentration (*X*) of the analyte. LLOQ was the lowest concentration on the calibration curve, and the signal-to-noise (S/N) ratio was >10.

### 2.9. Intra- and Inter-day Accuracy and Precision

Intraday values were evaluated within one day; inter-day accuracy and precision were performed in replicates of six in three consecutive days at three QC levels. The actual concentrations of the measured samples were determined by the calibration curve constructed on the same day and compared with the marked concentration. The accuracy was expressed as the relative error (RE%). The precision of the determination was described by the relative standard deviation (RSD%). For QC samples, the accuracy and precision were acceptable within ±15% and the LLOQ should be ±20%.

### 2.10. Matrix Effects and Recovery

The peak regions of blank plasma were compared with those of the spiked QC samples and normative samples at the same concentration (three QC concentrations in six replicates) to determine the matrix effect. The peak regions of diosgenin and IS extracted from plasma samples were compared with the peak regions of the pure normative sample, in which there was no extraction, to define the recoveries. Measurements of the extraction recoveries of three levels of diosgenin QC samples and IS were performed for six replicates.

### 2.11. Stability

QC samples in rat plasma at three concentrations were analyzed by assessing the stability of diosgenin under four conditions. Long-term stability was investigated by assaying QC samples kept at −20°C for 30 days. The short-term stability of QC samples was assessed for 8-h storage at 25°C. Freeze-thaw stability was determined by analyzing QC samples after three freeze-thaw cycles (−40°C to 25°C). Postpreparative stability was evaluated after a 24-h placement of QC samples in an autosampler at 4°C after preparation. When the accuracy and the precision were both within ±15% after the four tests, the stabilities were guaranteed.

### 2.12. Pharmacokinetic Study

Male Sprague-Dawley rats (*n* = 12) weighing 180–220 g were supplied by Beijing Vital River Laboratory Animal Technology Co., Ltd. (Beijing, China; license number SCXK2018-0006). According to the ARRIVE guidelines, all experimental rats were kept under appropriate conditions, including proper humidity, temperature, light, diet, and water. Before starting the experiments, the rats were domesticated for 10 days under laboratory conditions to minimize their suffering. All experimental procedures were performed according to the Animal Care and Use Committee of Chengde Medical University (ethical approval no. CDMULAC-20180410016; Chengde, China). The rats were divided into two groups and were orally administered 100 mg/kg diosgenin and ASD (equivalent to 100 mg/kg diosgenin) suspended in a 0.5% w/v CMC–Na aqueous solution.

The caged rats were fasted (water provided ad libitum) for 12 h before the experiment. Before and after single administration of diosgenin, 0.15 ml blood was collected from the inner canthus under 2.5% isoflurane anesthesia in a heparinized blood collection vessel. The blood collection time points were 0.083, 0.25, 0.5, 0.75, 1, 2, 3, 4, 6, 8, 12, 24, 36, 48, and 72 h. Plasma was obtained after centrifugation for 15 min at 15,000 rpm and then stored at –80°C until analysis.

The pharmacokinetic parameters were calculated using noncompartmental pharmacokinetics data analysis software of DAS 3.0 (Drug and Statistics software, Drug Clinical Research Center, Shanghai University of Traditional Chinese Medicine, Shanghai, China). Relative bioavailability (F) was calculated according to the following formula:

(1) $$\begin{array}{c} \text{F}\left(\text{\%}\right)=\frac{{\text{AUC}}_{\left(0⟶\infty \right)\left(\text{diosgenin ASD}\right)}}{{\text{AUC}}_{\left(0⟶\infty \right)\left(\text{diosgenin}\right)}}\times 100\text{\%}. \end{array}$$

## 3. Results and Discussion

### 3.1. Method Development

In vivo pharmacokinetics is very important for the development of new drugs, but there are few studies on the pharmacokinetics of diosgenin. When establishing a drug analysis method, the choice of the instrument, chromatographic column, mobile phase, and plasma sample extraction method will affect the sensitivity and accuracy of the method. GC and HPLC methods were mainly used to separate diosgenin, and liquid-liquid extraction was mostly used for the treatment of plasma samples. Liu Lijian employed a GC-MS method to determine the content of diosgenin in rats. Diosgenin was extracted with n-hexane-ethyl acetate (90 : 10, v/v), and LLOQ was 13 ng/ml and separation time >18 min [24]. In Xu Lina's study, analyses were carried out using an Agilent 1200 HPLC system, and diosgenin was extracted with n-hexane-ethyl acetate (90 : 10, v/v), with LLOQ being 10 ng/ml [27]. Liu Haifeng used an Agilent 1100 LC-MSD system including a binary infusion pump, G1312 A autosampler, and G1946D quadrupole mass spectrometer detector to detect diosgenin. Diosgenin in plasma samples was extracted with 3 ml of water-saturated ethyl acetate, LLOQ was 50 ng/ml, and the total analysis time was more than 10 min [37]. Wang Yang used a Waters 2695 HPLC coupled with a Micromass Quattro secondary mass spectrometer to detect the content of diosgenin in plasma samples. Diosgenin was extracted by isopropanol-ethyl acetate (10 : 90, v/v), and the separation time was >6 min [38]. These analytical methods have some limitations. The selective ion detection mode is used to determine the selectivity, which is easy to be interfered by complex matrices, resulting in false positive results. Moreover, the plasma processing method in all these studies was liquid-liquid extraction, and the operation process was cumbersome. UPLC-MS/MS combined technology using the MRM mode is a detection method with strong selectivity and high sensitivity. Compared with HPLC, UPLC has obvious advantages in terms of resolution and peak shape improvement. In addition, the use of UPLC short columns can shorten the separation time and improve work efficiency. Our research group has, for the first time, established a UPLC-MS/MS method to determine the concentration of diosgenin in rat plasma. Diosgenin was determined after methanol-mediated plasma protein precipitation. The separation was performed on the Phenomenex Kinetex XB C_18_ column (2.1 mm × 50 mm, 2.6 *μ*m) and eluted with a gradient mobile phase consisting of methanol (containing 0.1% formic acid)–0.1% aqueous formic acid. The equipment used was Waters AcQuity Ultra Performance LC (including binary pump and autosampler) in series with a Waters AcQuity TQ Detector triple quad pole mass spectrometer with an ESI source [39]. As the equipment was relatively old, the minimum detection limit was 10 ng/ml. In the present work, more advanced equipment was used to further optimize the strip, and a more sensitive and accurate method for the determination of diosgenin was established.

Evaluation of positive and negative ESI modes was performed. The results showed that the positive ESI mode yielded a stronger signal and better sensitivity than the negative ESI mode. After the optimization of various parameters, we evaluated samples as described in the Materials and Methods section, and the ion pairs of diosgenin and IS were detected at *m/z* 415.2⟶271.2 and *m/z* 295.2⟶277.1, respectively.

To improve resolution and peak shape, we optimized the chromatographic column, column temperature, and the types, proportions, elution modes, and flow rates of the mobile phase to obtain shorter analysis times and better results. The results showed that Waters ACQUITY UPLC BEH C_18_ (2.1 × 50 mm, 1.7 *μ*m), with a column temperature of 35°C and a flow rate of 0.3 ml/min, yielded the best resolution. For the mobile phase, we compared peak shapes of methanol-water, acetonitrile-water, and methanol-acetonitrile-water. The results showed that acetonitrile-water yielded sharper peaks than other solutions. At the beginning of the study, we attempted to analyze the gradient elution. However, the peaks of different proportions of the mobile phase showed bifurcation and severe trailing. In contrast, the isocratic elution resulted in a more stable signal, although the peaks showed trailing without formic acid. In a previous study, formic acid enhanced the ion signal and achieved better resolution and sharper peaks [40]. Finally, we selected isocratic elution with acetonitrile-water (including 0.3% formic acid) at a ratio of 80 : 20 (v/v), with a separation time of only 5 min (including separation, column cleaning, and equilibration time) as the optimal conditions. Compared with that in the existing literature, the separation method established in this work was easier to operate, and the analysis time was shorter.

The extraction of samples can affect the sensitivity and reliability of bioanalysis. To achieve acceptable recovery and matrix effects, plasma samples were processed using liquid-liquid extraction, protein precipitation, and solid-phase extraction columns. The plasma processing method with the solid-phase extraction column was complex, expensive, and did not produce satisfactory results; therefore, this method was eliminated. Liquid-liquid extraction with trichloromethane, ethyl acetate, trichloromethane-ethyl acetate (90 : 10, v/v), isooctane-isopropanol (80 : 20, v/v), methyl tert-butyl ethyl acetate (90 : 10, v/v), and other extracting solvents was used to process plasma. However, none of these solvents achieved acceptable recovery or matrix effects. In contrast, in this study, protein precipitation was finally chosen because of its velocity, simplicity, low matrix effects, and high recoveries. After evaluating several organic solvents, such as acetonitrile, methanol, and a mixture of methanol and acetonitrile with various proportions, methanol-acetonitrile (50 : 50, v/v) was selected as the precipitant because of its excellent performance. The plasma samples were diluted 4, 6, and 8 times with methanol-acetonitrile (50 : 50, v/v) for protein precipitation. The final 6-fold dilution was chosen because it provides satisfactory recovery rates, matrix effects, and acceptable sensitivity.

### 3.2. Method Validation

#### 3.2.1. Selectivity

**Figure 3:**
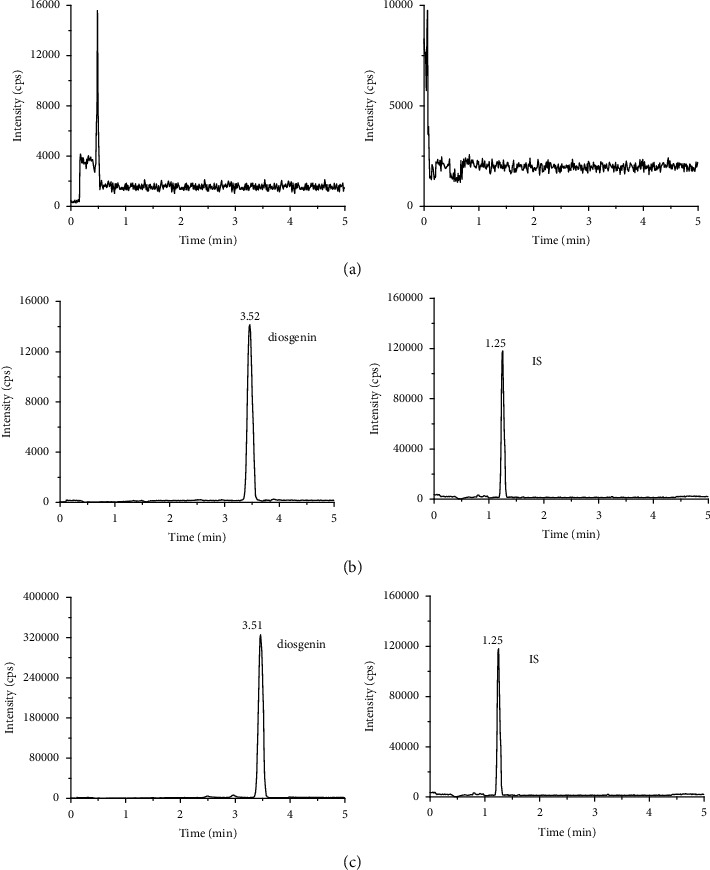
Representative chromatograms of diosgenin and IS in rat plasma samples. (a) A blank plasma sample; (b) a blank plasma sample spiked with diosgenin at LLOQ level and IS; (c) a rat plasma sample taken 12 h after oral administration of diosgenin (100 mg/kg) in rats.

Blank plasma samples from six rats were analyzed to determine selectivity. There was no obvious interference in representative chromatograms for diosgenin and IS (Figure 3) under the UPLC-MS/MS conditions, demonstrating the excellent selectivity of the approach.

#### 3.2.2. Linearity and LLOQ

The UPLC-MS/MS approach was used to measure the linear equation of the calibration curve, linear scope, and LLOQ of diosgenin. Based on the assay, excellent linearity was observed for diosgenin concentrations over the range of 0.5–1,500 ng/ml. The equation for the calibration curve was *Y* = 4,543.64436*X* + 16,5578 (*r* = 0.99752). The LLOQ of diosgenin was 0.5 ng/ml, and both the accuracy and precision were <20%, indicating the approach was sufficiently sensitive. It can be seen from the results that the linear scope in these experiments was 0.5 ng-1,500 ng/ml, and LLOQ could be 0.5 ng/ml. However, in actual pre-experiments, the minimum concentration of all samples was higher than 2 ng/ml, and thus, the low concentration point of QC samples was determined to be 5 ng/ml when the method was established.

#### 3.2.3. Matrix Effects, Recovery, Accuracy, and Precision

As shown in Table 1, at the three QC concentrations, the matrix effects of diosgenin measurement on rat plasma were from 92.36% to 93.22%, whereas those on IS were 95.56%. The RSD% in these samples was <5.0%. Hence, there were no significant matrix effects for diosgenin. The extraction recoveries of diosgenin in plasma ranged from 85.81% to 100.27%, and it was 98.29% for IS. The RSD% in these samples was <7.0%. According to these results, the sample processing methods used were appropriate.

Table 2 shows the accuracy and precision results of diosgenin for three consecutive days. The absolute RE% values for intra- and inter-day accuracy were <10%, and the precision values were <15%, with a maximum RSD% of 6.91%. All of the outcomes were in good agreement, indicating that the approach had excellent reproducibility. Hence, the approach was appropriate for determining the components in plasma samples.

#### 3.2.4. Stability

Under the storage conditions, diosgenin remained stable in plasma, and the results are summarized in Table 3. No significant loss of diosgenin occurred at –20°C for 30 days, at 25°C for 8 h, under three freeze-thaw cycles, and at 4°C in an autosampler for 12 h. Overall, this approach was suitable for researching the pharmacokinetic profiles of diosgenin in rats.

#### 3.2.5. Pharmacokinetic Analysis

**Figure 4:**
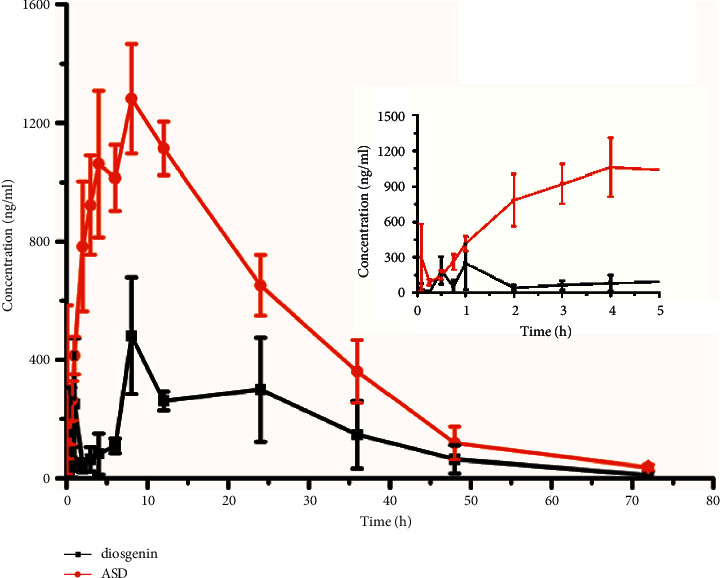
Plasma concentration-time profiles following oral administration of diosgenin and its ASD (mean ± SD, n = 6).

A UPLC-MS/MS approach was set up with tanshinone IIA as IS and was then used for pharmacokinetic analysis of diosgenin ASD in rats after oral administration. The pharmacokinetic profiles of diosgenin and diosgenin ASD were calculated using a noncompartmental model.  Figure 4 and Table 4 show the average plasma concentration-time curves and major pharmacokinetic coefficients of diosgenin and diosgenin ASD, respectively.

The poor solubility of diosgenin in water is one of the important reasons for the low efficacy of saponins. ASD is one of the most commonly used technologies to improve the dissolution rate of insoluble drugs. Therefore, in this study, the bioavailability of diosgenin was enhanced by preparation using ASD, providing a basis for the clinical application of this compound. As shown in Figure 4 and Table 4, compared with diosgenin, the *in vivo* performance of diosgenin ASD was significantly increased. The AUC_0–t,_ and *C*_max_ of ASD were 42,972.58 ± 6,864.96 *μ*g/l·h and 1,281.65 ± 262.08 *μ*g/l, respectively; these values were 4.61- and 2.67-fold higher than those of diosgenin, resulting in relative bioavailability of 453.12%. The *T*_max_ of diosgenin ASD (10.07 ± 3.65 h) was slightly longer than that of diosgenin (8.73 ± 5.74 h), although no significant differences were observed. The polymer may postpone the absorption of diosgenin, although significant effects were observed. The apparent volume distribution (Vz/F) and the clearance rate (CLz/F) of ASD were evidently lower than those of diosgenin, 5.23-fold, and 3.87-fold, respectively. Thus, these findings showed that ASD had a significantly longer retention time *in vivo* than diosgenin and that the bioavailability of diosgenin was enhanced for diosgenin ASD prepared using the coprecipitation approach.

## 4. Conclusion

After oral ingestion, diosgenin levels were determined in rats using a simple, quick, and sensitive UPLC-MS/MS approach. Protein precipitation was used to process the samples, and separation was carried out using chromatography for 5 min. The approach was validated by pharmacokinetic analysis. According to the pharmacokinetic data, the plasma levels of diosgenin ASD were significantly higher than those of diosgenin after oral administration. This basic approach provided insights into the appropriate selection of the dosage form for diosgenin, which may facilitate the application of this drug.

## Figures and Tables

**Table 1:** Extraction recovery and the matrix effect of diosgenin and IS on plasma determined by UPLC-MS/MS (*n* = 6).

	Concentration (ng/ml)	Extraction recovery		Matrix effect	
		Mean (%)	RSD (%)	Mean (%)	RSD (%)
Diosgenin	5	85.81	5.83	93.22	3.63
	50	86.66	6.49	92.36	4.12
	1000	100.27	5.75	93.07	3.47

IS	42	98.29	6.09	95.56	2.28

**Table 2:** Accuracy and precision of diosgenin in plasma determined by UPLC-MS/MS.

Indicated concentration (ng/ml)	Accuracy (RE%,*n* = 18)	Precision (RSD%)	
		Intraday (*n* = 6)	Interday (*n* = 18)
LLOQ (0.5)	−6.54	6.91	2.84
5	4.71	4.32	3.68
50	−4.78	3.27	2.56
1000	2.99	1.42	1.25

**Table 3:** Stability of diosgenin for the determination of the three concentrations in rat plasma (*n* = 6).

	Concentration (ng/ml)		RSD/%
	Indicated	Measured	
Short-term stability at 25°C for 8 h	5	5.41 ± 0.33	6.09
	50	50.23 ± 2.56	5.10
	1000	1000.88 ± 44.34	4.43

Long-term stability at −20°C for 30 days	5	5.99 ± 0.28	4.67
	50	50.02 ± 3.72	7.43
	1000	1001.65 ± 42.42	4.23

Freeze-thaw stability, three cycles	5	5.98 ± 0.35	5.85
	50	51.69 ± 4.36	8.43
	1000	1002.9 ± 54.44	5.42

Autosampler stability at 4°C for 24 h	5	5.32 ± 0.37	6.95
	50	50.59 ± 3.69	5.42
	1000	1001.01 ± 62.63	5.42

**Table 4:** Pharmacokinetic parameters following oral administration of diosgenin and its ASD (mean ± SD, *n* = 6).

Parameter	Diosgenin	ASD
AUC_0-t_ (*μ*g/l*∗*h)	9,302.27 ± 5,013.38	42,972.58 ± 6,864.96^*∗∗*^
AUC_0-∞_ (*μ*g/l*∗*h)	9,598.01 ± 5,151.91	43,490.51 ± 7,648.62^*∗∗*^
MRT_0- t_ (h)	22.77 ± 5.55	21.05 ± 4.30
*t*1/2*z* (h)	12.33 ± 3.02	10.67 ± 2.59
Tmax (h)	8.73 ± 5.74	10.07 ± 3.65
CL_z/F_ (L/h/kg)	12.41 ± 6.13	3.20 ± 0.63^*∗∗*^
*V*_z/F_ (L/kg)	231.16 ± 161.21	44.18 ± 6.78^*∗∗*^
Cmax (*μ*g/l)	480.09 ± 121.31	1,281.65 ± 262.08^*∗∗*^

Compared with diosgenin, ^*∗*^*P* < 0.05,^*∗∗*^*P* < 0.01.

## Data Availability

All data generated or analyzed during this study are included in this published article.
